# Validated predictive modelling of the environmental resistome

**DOI:** 10.1038/ismej.2014.237

**Published:** 2015-02-13

**Authors:** Gregory CA Amos, Emma Gozzard, Charlotte E Carter, Andrew Mead, Mike J Bowes, Peter M Hawkey, Lihong Zhang, Andrew C Singer, William H Gaze, Elizabeth M H Wellington

**Affiliations:** 1 School of Life Sciences, University of Warwick, Coventry, UK; 2 NERC Centre for Ecology & Hydrology, Wallingford, UK; 3 Applied Statistics Group, Department of Computational and Systems Biology, Rothamsted Research, Hertfordshire, UK; 4 Health Protection Agency, West Midlands Public Health Laboratory, Heart of England NHS Foundation Trust, Birmingham, UK; 5 Institute of Microbiology and Infection, Biosciences, University of Birmingham, Birmingham, UK; 6 European Centre for Environment and Human Health, University of Exeter Medical School, Knowledge Spa, Royal Cornwall Hospital, Truro, UK

## Abstract

Multi-drug-resistant bacteria pose a significant threat to public health. The role of the environment in the overall rise in antibiotic-resistant infections and risk to humans is largely unknown. This study aimed to evaluate drivers of antibiotic-resistance levels across the River Thames catchment, model key biotic, spatial and chemical variables and produce predictive models for future risk assessment. Sediment samples from 13 sites across the River Thames basin were taken at four time points across 2011 and 2012. Samples were analysed for class 1 integron prevalence and enumeration of third-generation cephalosporin-resistant bacteria. Class 1 integron prevalence was validated as a molecular marker of antibiotic resistance; levels of resistance showed significant geospatial and temporal variation. The main explanatory variables of resistance levels at each sample site were the number, proximity, size and type of surrounding wastewater-treatment plants. Model 1 revealed treatment plants accounted for 49.5% of the variance in resistance levels. Other contributing factors were extent of different surrounding land cover types (for example, Neutral Grassland), temporal patterns and prior rainfall; when modelling all variables the resulting model (Model 2) could explain 82.9% of variations in resistance levels in the whole catchment. Chemical analyses correlated with key indicators of treatment plant effluent and a model (Model 3) was generated based on water quality parameters (contaminant and macro- and micro-nutrient levels). Model 2 was beta tested on independent sites and explained over 78% of the variation in integron prevalence showing a significant predictive ability. We believe all models in this study are highly useful tools for informing and prioritising mitigation strategies to reduce the environmental resistome.

## Introduction

Understanding the drivers of antibiotic resistance is essential if we are to combat the problem of rapidly emerging multi-resistant pathogens. It has been well established in the clinic that increasing antibiotic usage escalates resistance levels (McGowan, 1983), yet it is less clear how current usage both in veterinary and human medicine has had an impact on the environmental resistome (Wellington *et al.*, 2013). Antibiotic resistance is naturally present in environmental bacteria, therefore all environments will have a base level of resistance capable of being selected for by antibiotic residues, detergents and heavy metals (D'Costa *et al.*, 2011; Knapp *et al.*, 2011; Forsberg *et al.*, 2012). Examples of anthropogenic inputs include wastewater-treatment plant (WWTP) effluent, which increases prevalence of clinically important resistant bacteria and resistance genes (Amos *et al.*, 2014a, 2014b), agricultural pollution where antibiotic-resistant bacteria reach the environment via animal faeces and slurry application (Chee-Sanford *et al.*, 2001; Kay *et al.*, 2005; Byrne-Bailey *et al.*, 2009), and finally detergents found in industrial effluent that co-select for class 1 integrons (Gaze *et al.*, 2005). Class 1 integrons are genetic elements that routinely contain mobile antibiotic and biocide-resistance genes (Stokes and Hall, 1989), which have been found in a wide range of polluted environments such as sewage-sludge-amended soil (Gaze *et al.*, 2011) and WWTP effluent (Stalder *et al.*, 2014). Class 1 integrons are capable of integrating gene cassettes into a variable region; to date they are over 130 gene cassettes conferring a range of antibiotic-resistant phenotypes, thus the presence of a class 1 integron gives the bacteria the ability to become resistant to a range of antibiotics (Partridge *et al.*, 2009). In this study, we hypothesise that class 1 integron prevalence can be used as a proxy for antibiotic resistance.

Research to date has primarily focused on analysing the effects of single variables on the environmental resistome, without the collection of metadata; although one study has attempted to model the associations between environmental variables and resistance gene prevalence, it did not consider water chemistry, rainfall or season (Pruden *et al.*, 2012). Our study is the first to integrate point (WWTPs) and diffuse (landscape) sources of pollution plus spatial, temporal, climatic and water-chemistry variables into a predictive model.

**Figure 1:**
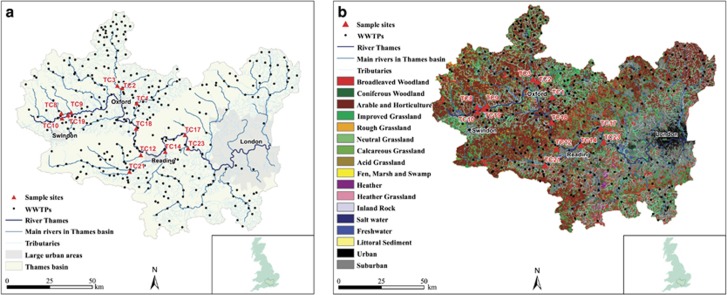
(**a**) Map illustrating the Thames Watershed in Oxfordshire, sampling sites and WWTPs. Site names are an extension of a previously defined naming system. (**b**) Map illustrating the Thames Watershed in Oxfordshire with associated land covers extracted from the LCM2007 (Morton *et al.,* 2007).

Our aim was to collect data on class 1 integron prevalence and β-lactam resistance in the Thames river catchment, integrate environmental metadata and produce a predictive model attributing sources of resistance. Class 1 integron prevalence was determined over 13 sites across the Thames river basin (Figure 1) in a longitudinal study with viable counts of third-generation cephalosporin (3GC)-resistant coliforms used to verify correlation of class 1 integron prevalence with phenotypic antibiotic resistance. Geospatial and chemical analyses allowed the development of mathematical and statistical models. Model 1 was mathematical and consisted of three submodels built up from varying levels of WWTP complexity. Models 2 and 3 were statistical, accounting for variations in WWTPs, land cover, weather and temporal changes (Model 2) and variations in river water chemistry (Model 3). A series of simulations based on these models were used to evaluate the impact of WWTP type, size and distance from sample site combined with the surrounding land cover, weather, temporal changes and river water chemistry on the environmental resistome.

## Methods

### Sampling

Triplicate sediment cores were taken for microbial analysis; in tandem water samples were taken for chemical analysis at the same site. Sediment cores were taken to an approximate depth of 10 cm and mixed thoroughly. For water samples grab samples were acquired in 1000-ml borosilicate brown glass bottles at the end of a 1.5-m-long sampling rod taken from the fast moving portion of the river at each location. Samples were stored at 4 °C and processed within 24 h of collection. A total of 13 sites were visited (Figure 1) in May, August and November in 2011 and in February 2012. Site TC19 missed the November and February measurements, and TC3 missed the May 2011 measurement due to extremely high water levels. Influent (1 litre) and effluent (1 litre) were collected as grab samples, in duplicate, from three WWTPs of different types.

### Prevalence of class 1 integrons

DNA was extracted from each sediment core (0.5 g) and from the sewage samples within 24 h of sample collection using Fast DNA extraction kit (MP Biomedicals, Solon, OH, USA). For both influent and effluent, 1 litre of sample was filtered through a 0.2-μm cellulose nitrate membrane. Filters were used directly for extraction using a modified Fast DNA extraction method (MP Biomedicals), where a 0.5 g of filter was used for extraction of DNA. Power SYBR Green Mastermix (Applied Biosystems, Calrsbad, CA, USA) was used with primers Int1F2 5′-TCGTGCGTCGCCATCACA-3′ and Int1R2 5′-GCTTGTTCTACGGCACGTTTGA-3′ as previously described for the detection of class 1 integrons by qPCR (Gaze *et al.*, 2011). Quantification of the 16S rRNA gene was performed using Power SYBR Green Mastermix (Applied Biosystems) with primers 16S1369f 5′-CGGTGAATACGTTCYCGG-3′ and 16S1492r 5′-GGWTACCTTGTTACGACTT-3′ as published; class 1 integron prevalence was estimated as the ratio of number of class 1 integrons to the number of 16S rRNA genes (Gaze *et al.*, 2011).

### Plate counts

Viable counts were performed for numbers of 3GC-resistant coliforms for sites in August and November, 2011 and in February 2012 as previously described (Amos *et al.*, 2014a). In brief 1 g of sediment was taken and resuspended in 9 ml of PBS buffer. Chromocult coliform agar (Merck Biosciences Ltd, Nottingham, UK) was prepared in accordance with the manufacturer's instructions and amended with cefotaxime (2 mg/l). Downstream and upstream samples were plated (200 μl) in triplicate before incubating for 24 h at 30 °C. Viable plate counts were taken; blue colonies indicated presumptive *Escherichia coli* and pink colonies indicated other coliforms. Reference strains of *E. coli*, *Klebsiella oxytoca*, *Citrobacter freundii*, *Pseudomonas fluorescens* and *Aeromonas media* were used to evaluate the performance of Chromocult at 30 °C.

### Geospatial analyses and rainfall collection

**Figure 2:**
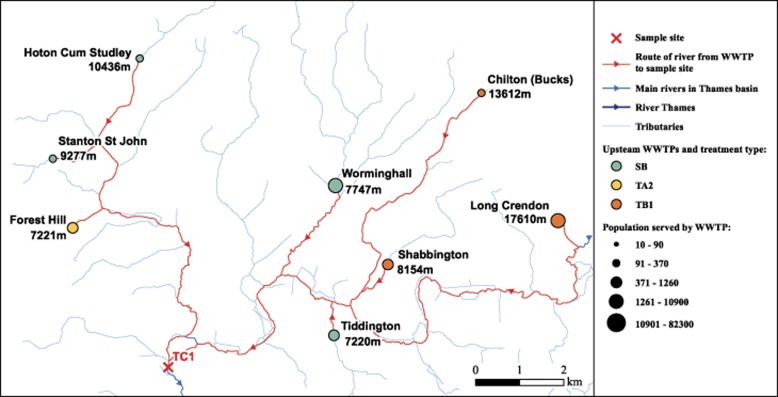
Example of geospatial analyses conducted at sampling sites. Map illustrating distances from all WWTPs within a 10-km buffer, which drained into the sampling site TC1.

All geospatial analyses were performed using ArcGIS v.10 (ESRI, Aylesbury, UK). Distances from all WWTPs (within a 10-km buffer) to sampling sites were calculated using the distance followed by the river course (Figure 2; Supplementary Figure 1). The percentage area of land cover within a 2-km buffer around each sampling site was calculated using the Centre for Ecology and Hydrology Land Cover Map 2007 (Morton *et al.*, 2007). Precipitation data were taken from a world weather database (http://www.tutiempo.net/en/; last accessed 6 January 2014).

### Statistical analyses

All collected data were tested for normality using measurements of kurtosis and skewness followed by a Shapiro–Wilk or Kolmogorov–Smirnov test for normality using IBM Statistics SPSS 21 (IBM, Portsmouth, UK). Non-normally distributed data were log transformed or square root transformed as appropriate to create a normally distributed data set. Correlation analyses between class 1 integron prevalence and chemical analyses, and between class 1 integron prevalence and landscape covers were performed using the Pearson's product-moment correlation coefficient with a subsequent determination of the significance from 0 (Genstat, 15th edition, VSN international, Hemel Hempstead, UK). For comparison of means Student's *t-*tests, analysis of variances and *post hoc* Tukey's honest significant difference tests were performed using IBM Statistics SPSS 21 (IBM). Multiple linear regression analyses, including stepwise variable selection approaches, were performed using GenStat. Models relating antibiotic resistance to the impact of WWTPs were fitted using a general non-linear model convergence process in GenStat, with convergence achieved using the Newton–Raphson algorithm and minimising the sum of squared deviations between the observed and modelled mean class 1 integron prevalence. Predictions and simulations of the impacts of different patterns of land use and locations of WWTPs were achieved using the predictive function for both linear and non-linear models in GenStat.

### Analytical chemistry

Chemical analyses of river water samples were performed as part of the Centre for Ecology and Hydrology Thames Initiative (Bowes *et al.*, 2012); alkalinity, pH, soluble reactive phosphorus (SRP), total dissolved phosphorus (TDP), total phosphorus (TP), ammonium (NH_4_), dissolved reactive silicon (Si), chlorophyll a (chla), fluoride (F), chloride (Cl), nitrite (NO_2_), bromine (Br), nitrate (NO_3_), sulphate (SO_4_), total dissolved nitrogen (TDN), sodium (Na), potassium (K), calcium (Ca), magnesium (Mg), boron (B), iron (Fe), manganese (Mn), zinc (Zn), copper (Cu) and aluminium (Al) were all analysed using published methods (Bowes *et al.*, 2012).

## Results

### Class 1 integron prevalence in River Thames basin

**Figure 3:**
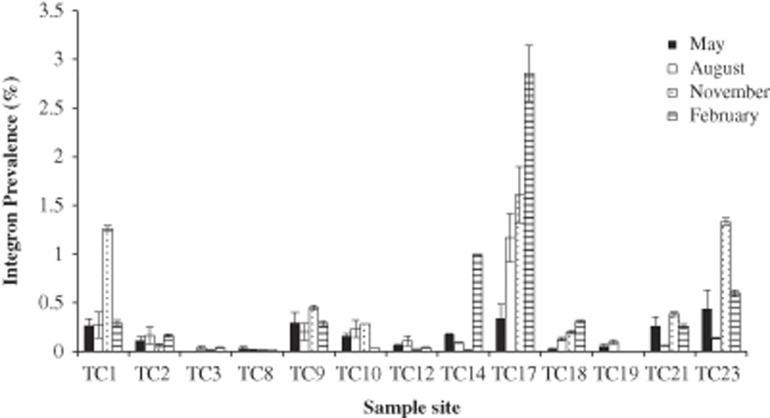
Class 1 integron prevalence taken from 13 sites across the Thames river basin at four time points. Class 1 integron prevalence was calculated as the ratio of *intI1* genes to 16S rRNA genes expressed as percentage. TC19 was not sampled in February or May, and TC3 was not sampled in May. Error bars are ±s.e.m. of three biological replicates.

There was a significant difference in class 1 integron prevalence between different sites in the River Thames basin (Figure 3; analysis of variance, *F*=6.845; *P*<0.001), these could be grouped into five homogenous subsets following a *post hoc* Tukey's honest significant difference test (Table 1). Class 1 integron prevalence was temporal with significant differences between samples from November and February compared to samples from May and August (*t-*value 3.11; *P* <0.001), potentially indicative of seasonal change.

### Geospatial analyses

Geospatial analyses were conducted allowing for the construction of 13 maps illustrating the size and distance of upstream WWTPs within a designated 10-km buffer (Figure 2; Supplementary Figure 1). The surrounding land covers were calculated as percentages (Supplementary Table 1).

### Relationship between surrounding land cover and class 1 integron prevalence

A correlation matrix was constructed using the Pearson's product-moment correlation to investigate association between landscapes (a marker of potential land use) as classified by the LCM2007, and class 1 integron prevalence (Supplementary Table 2). There were clear landscape types that positively correlated with class 1 integron prevalence such as: Acid Grassland, Heather Grassland, Freshwater, Suburban and Urban land uses (Pearson's coefficients 0.4674, 0.5533, 0.3976, 0.4053 and 0.3222, respectively; *P*<0.01 in all cases except the last (*P*=0.0239)), indicative of land uses that may be associated with resistance. Landscape types were also negatively correlated with class 1 integron prevalence, such as Rough Grassland and Arable/Horticulture (Pearson's coefficients −0.3422 (*P*=0.0161) and −0.386 (*P*<0.01), respectively).

### Model construction for impact of WWTPs on antibiotic resistance in rivers

In order to analyse the impact of WWTP effluent on antibiotic resistance in the river sediment it was necessary to account for the considerable diversity of geospatial characteristics between sampling sites (Figure 2; Supplementary Figure 1). To capture this diversity a model was constructed (Model 1) based on a series of testable hypotheses and assumptions.

### Model 1A, impact of an individual WWTP at a given sampling site

 


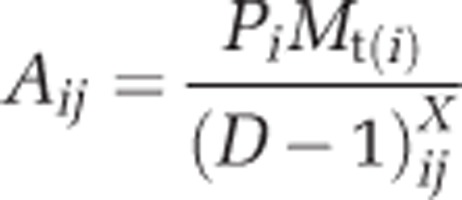


Where, *i* = specific WWTP at site, *j* = sampling site, t = type of WWTP, *A* = impact of WWTP *i* on class 1 integron prevalence at sampling site *j*, *P* = population equivalent, *M* = loading of WWTP *i* of type t, *D* = distance of WWTP *i* from site *j* and *X* = parameter that defines how the impact of a WWTP decays with distance to the sampling site.

Hypothesis 1: Class 1 integron prevalence is a marker of antibiotic resistance; higher class 1 integron prevalence is indicative of elevated resistance load.

Hypothesis 2: Different types of WWTP differ in the influent received and their efficacy for removal of antibiotic-resistant bacteria and selective residues. Assuming there are only seven types of WWTP as set out in the Water Services Regulation Authority scheme (Williams *et al.*, 2008) with no variability within each treatment type, the impact a treatment type has on *A* will be equal to *M*.

Hypothesis 3: The effect of effluent on *A* will decay with distance (*D*) due to dilution of effluent; decay rate will be equal to the exponent *X*.

Assumption 1: The size of the WWTP will be reflected by the population equivalent. Population equivalent has previously been used as the standard method for inferring the size of a WWTP (*P*) (De Feo *et al.*, 2013).

### Model 1B, summarising the effect of all WWTPs at a given site

 


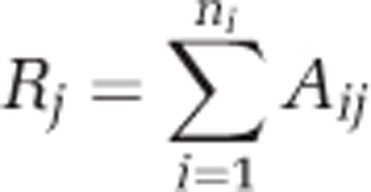


Where, *R* = total impact of WWTPs on class 1 integron prevalence at a single point in a river.

Assumption 2: Total impact of WWTPs on class 1 integron prevalence at a single point in a river will be equal to the sum of the impact of each individual WWTP.

### Model 1C, class 1 integron prevalence calculation

 


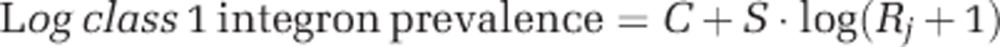


Where, *C* = indigenous level of antibiotic resistance, *R* = total impact of WWTPs on class 1 integron prevalence at a single point in a river and *S* = rate of increase in class 1 integron prevalence with increasing WWTP impact.

### Testing hypothesis 1: class 1 integron prevalence as a proxy for phenotypic antibiotic resistance

Viable counts of coliforms resistant to the clinically important 3GC antibiotics were made at selected sites where class 1 integron prevalence was measured. There was a highly significant positive correlation between class 1 integron prevalence and numbers of 3GC-resistant coliforms (Pearson's coefficient 0.8415; *P*<0.001). A linear regression was used as a secondary measure to analyse the relationship, with class 1 integron prevalence explaining 69% (*P*<0.001) of the variability (adj-*R*^2^=0.69) between numbers of 3GC-resistant coliforms, validating the approach to use class 1 integron prevalence as a marker of resistance.

### Testing hypothesis 2: the incoming influent, treatment and outgoing effluent varies between different types of WWTP

Triplicate samples of influent and effluent were taken from three different types of WWTP and analysed for class 1 integron prevalence (Supplementary Figure 2). Cholsey, a Tertiary Biological 2 (TB2)-type WWTP reduced the class 1 integron prevalence by a third; Ascot, a Secondary Activated (SA) WWTP increased the class 1 integron prevalence by over fourfold; and Benson, a Secondary Biological (SB) type reduced the class 1 integron prevalence by over a half. Results confirm the hypothesis that the influent, treatment and outgoing effluent vary greatly between WWTPs. This was further tested by convergence modelling using the observed data across the 13 sites in the river catchment, which received effluent from six different types of WWTP (Table 2).

### Testing hypothesis 3 and calculating model 1 parameters

A previous study of distance in relation to impact of WWTP on antibiotic resistance load conducted by Czekalski *et al.* (2014) reported that distance had a negative impact on the antibiotic resistance load and this relationship was non-linear. In order to expand on this and measure the exponent for *X* as well as measure the variability of *M*, thus producing values for the parameters in model 1, convergence modelling was used (with convergence reached using the Newton–Raphson algorithm). Convergence modelling was performed by utilising the results of the geospatial analyses of the 13 sites (giving values for *D* and *P*) and entering known values for log class 1 integron prevalence across the 13 sites into Model 1, then implementing general non-linear model convergence and general non-linear regression analysis (Table 2). From this analysis, we calculated that parameter *M*, which had a large range of values between different treatment types demonstrated by Secondary Activated (SA) at 0.9115 compared to Tertiary Biological 2 (TB2) at 0.2722. Modelled values for *M* were in agreement with observed measurements of class 1 integron removal, for example secondary activated plants have a larger impact on environment-resistance levels than TB2 plants. Values for other WWTPs as calculated by general non-linear model convergence and general non-linear regression analysis (Table 2) are the modelled efficacy of antibiotic resistance load reduction of all seven types of WWTPs relative to each other.

The value of *X* determined by convergence reached using the Newton–Raphson algorithm was 0.3875, indicating that the impact of WWTP effluent on antibiotic resistance in the sediment will decay at a rate equal to (*D*−1)^−0.3875^.

Although in Model 1 the term *C* was initially unknown, previous studies of unpolluted soils have measured class 1 integron prevalence of 0.001–0.002% (Gaze *et al.*, 2011), which is similar to the antilog value of *C* (0.017%), validating the methodology of excluding WWTPs >10 km from sampling sites.

Model 1C was used to generate predicted class 1 integron values for each of the 13 sites, which was regressed against the observed values collected across the four seasons. The resulting coefficient of determination (adj-*R*^2^) was 0.495 (*P*>0.001), therefore measuring the overall impact of all WWTPs on antibiotic resistance at a single site (*R*), explains 49.5% of the variance in antibiotic-resistance levels.

### Model 2: evaluating the impact of WWTPs in combination with land cover, temporal changes and rainfall

Owing to the previous associations of resistance with land cover, the potential for temporal changes in class 1 integron prevalence and the calculated impact of WWTPs (*R*) on resistance it was deemed appropriate to incorporate all factors into a multiple linear regression to form a ‘super model' (Model 2). Input variables were percentage land cover, *R* as calculated from Model 1 and log rainfall. To simulate temporal changes all factors were allowed to vary between four time points by using regression with groups. Model 2 was identified from an all-subsets regression analysis and a forward and backwards stepwise variable selection analysis (Table 3).

**Figure 4:**
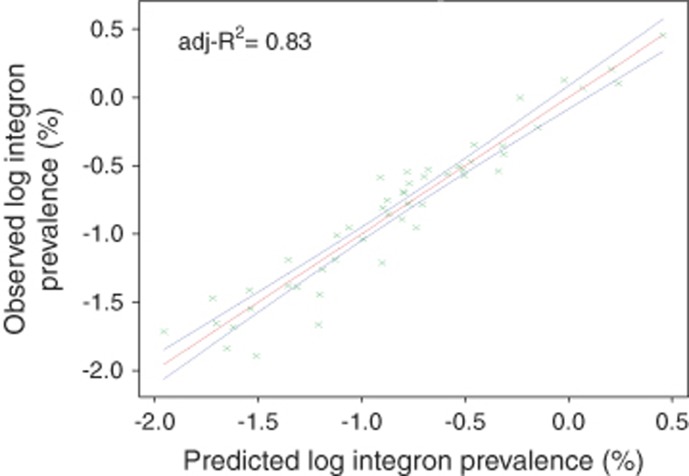
Output from model 2, which explains 82.9% of the variation in antibiotic-resistance levels in the River Thames basin. Red line is predicted values, blue line is 95% confidence levels.

Model 2 explained 82.9% of the variance of log class 1 integron prevalence at a single point in a river at any season (*P*>0.001; Figure 4). Many variables had associations with resistance depending on time of year, for example, Heather Grassland had a strong negative effect for most of the year except in winter months when a strong positive effect was conferred. Precipitation also had an impact on resistance, this varied depending on the surrounding land covers. A heavy rainfall at a point surrounded by neutral grassland would elevate resistance levels, where as a heavy rainfall at a point surrounded by Woodland would decrease resistance levels. Using values for precipitation falling on the day before sampling resulted in the best model fit.

### Assessing the predictive ability of Model 2

**Figure 5:**
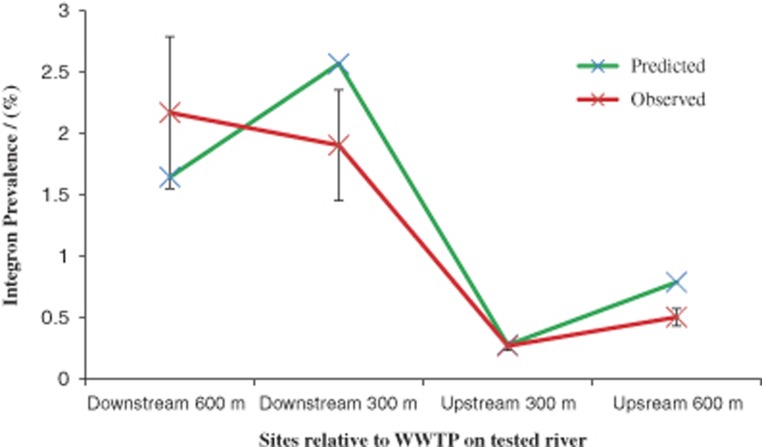
Comparison of average predicted values for class 1 integron prevalence in a river from the Midlands based on model 2, compared to observed values for class 1 integron prevalence. Samples were taken from upstream and downstream of a WWTP at 300- and 600-m intervals (Amos *et al.,* 2014a). Error bars for observed are based on ±s.e.m. of three biological replicates.

The predictive ability for Model 2 was tested on four sites using known class 1 integron prevalence values from a prior study in central England (both temporally and spatially independent from the River Thames samples). Data for the explanatory variables were available (Amos *et al.*, 2014a). Model 2 explained 78.4% of the antibiotic-resistance levels in the four sites independent from the River Thames (Figure 5), this is similar to the 82.9% calculated from the data set on which the model was based, validating the model as accurate and transferable. Estimates ranged between 64.1% and 131.7% of the mean observed target.

### Simulating impact of a WWTP on a clean site

The explanatory variable with the highest *t*-value in Model 2 was *R* (total impact of WWTPs at a single point in a river). To evaluate the role WWTPs play in levels of antibiotic resistance in aquatic systems, we simulated the impact of a large (850 000 population equivalent) activated sludge-treatment plant (similar to those which serve UK cities) on the clean site TC8. The initial estimate of impact of the effluent was a 200-fold increase in class 1 integron prevalence (0.01–2.44%). A prevalence of 0.77% was predicted 10 km downstream of the WWTP, which is still a 65-fold increase compared to no WWTP.

### Simulating an intervention strategy of reducing the impact of WWTPs on a site with high antibiotic-resistance levels

Model 2 was used to predict the influence of reducing the impact of WWTPs (*R*) on a site with a high level of antibiotic resistance (TC17). Simulations were performed by changing the actual *R* value; a reduction in *R* at site TC17 to the average value of *R* in the data set would reduce the class 1 integron prevalence from 1.86% to 0.93%. Reducing the value of *R* equivalent to the lowest in the Thames data set would reduce the class 1 integron prevalence to 0.24%, which would be the fifth lowest in the catchment area. This emphasises the significance of *R* in Model 2, which in turn demonstrates the large impact WWTPs have on environmental antibiotic-resistance levels.

### Correlations between water quality parameters and antibiotic-resistance levels

Relationships between water quality parameters and class 1 integron prevalence were investigated using Pearson's correlation coefficients (Supplementary Table 3). Correlation matrices revealed class 1 integron prevalence significantly correlated with levels of zinc, total phosphorous, total dissolved phosphorous, silicon, manganese, potassium and copper (Pearson's coefficient *P* <0.05 in all cases). Several chemicals co-correlated with each other.

### Model 3: water quality parameters as markers for antibiotic resistance

To identify variables with explanatory power for class 1 integron prevalence multiple linear regression analyses were performed to develop Model 3. The best model was identified from an all-subsets regression analysis and a forward and backwards stepwise variable selection. The output from this regression (Supplementary Table 4) was used to derive model 3, which could explain 71.4% of the variance in class 1 integron prevalence. Total phosphate had the most significant contribution to the model denoted by the highest *t*-value (4.56). Many variables that showed significant correlations with class 1 integron prevalence were not significant in the best multiple linear regression model due to strong correlations between the explanatory variables.

### Model 3

 


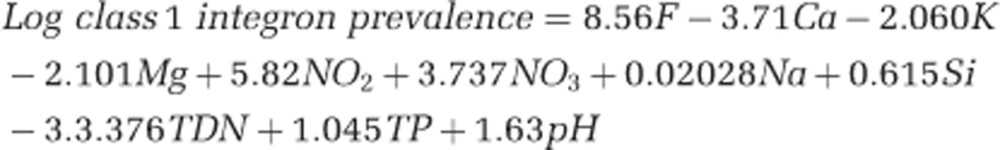


An equation for calculating log class 1 integron prevalence derived from Supplementary Table 4.

## Discussion

We report here two models (Models 2 and 3; Model 1 was integrated into Model 2) to predict antibiotic-resistance levels within a river catchment that require no prior knowledge of antibiotic-resistance levels or bacterial communities. The predictive models rely on routinely collected data, such as landscape covers, precipitation levels or water quality parameters thereby facilitating application to other river catchments. Moreover this is the first study to identify, attribute and quantify this range of variables associated with antibiotic resistance, and has successfully explained 83% of the variance of resistance levels in the aquatic environment. We demonstrated that resistance is escalated by the abiotic impacts of WWTP effluent and Urban and Suburban land covers, as well as being negated by biotic factors, such as large areas of Coniferous Woodland and Rough Grassland, with prevailing weather patterns also impacting on resistance loads. Model 2 was beta tested on four sites both spatially and temporally independent of the data set on which the model was derived. The significant predictive power of Model 2 in these sites both validated the model as a predictive tool and supports conclusions drawn from the model attributing drivers of resistance in the environment.

Data collected for class 1 integron prevalence and antibiotic-resistant coliforms in the River Thames catchment illustrates the temporal and spatial variability of resistance gene prevalence in river systems. For the first time, we demonstrate the utility of class 1 integrons as a proxy for antibiotic resistance in the environment, with significant correlation between integron prevalence and 3GC resistance across three different time points and several sample points. This is likely due to the aggregation of resistance genes, which occurs on many plasmids with class 1 integrons present (Amos *et al.*, 2014a; Tacao *et al.*, 2014). There were significant temporal changes in class 1 integron prevalence and model fitting was optimal when there were four separate time points. Accounting for prior immediate rainfall (previous day) significantly increased the accuracy of the model, yet accounting for temporal change also improved the fit of the model potentially suggesting there is both a long and short term impact of prevailing weather. Resistance patterns in a temperate climate may reflect oscillations in rainfall. Significant seasonal change in antibiotic resistance gene transport between wet and dry seasons has previously been reported in other climates (Luo *et al.*, 2010; Knapp *et al.*, 2012).

Model 1 demonstrated that WWTPs were mainly responsible for variance in antibiotic-resistance levels in rivers (explaining 49.5% of variance), which supports growing evidence that WWTPs introduce and select for large numbers of antibiotic-resistant bacteria (Amos *et al.*, 2014a). Model 1 illustrates the key determinants of a WWTP, which are the size of the plant, type and distance from the site of sampling. Czekalski *et al.* (2014) observed that distance of WWTPs from sample points impacts the levels of resistance downstream in a non-linear manner; we agree and proved that a rate of *X*^−0.385^ describes this non-linearity. These observations have profound implications for the lasting impacts of WWTP effluent as the model predicts increased persistence of resistance genes downstream in rivers. Treatment type also played a key role, supporting previous studies that have demonstrated the variability in resistance gene removal across treatment plants (Chen and Zhang, 2013). WWTPs using tertiary activated sludge as a way of treatment had the lowest impact on the rivers as demonstrated by Model 1. The difference between secondary activated sludge and tertiary activated sludge 1 indicated up to a 100-fold decrease in impact on class 1 integron prevalence in the river when using the tertiary treatment. Model 2 illustrated through simulations that by reducing the impact of WWTPs (such as through an upgrade in treatment type), it is possible to reduce the antibiotic resistance load at highly polluted sites, thus highlighting a potential role Model 2 could play in designing mitigation strategies to reduce load in river catchments. A mechanism for the reduction of antibiotic resistance may be through filtration (often employed at the tertiary treatment stage), which has been previously suggested to reduce antibiotic resistance load in effluent (Munir *et al.*, 2011; Stalder *et al.*, 2013).

Previous studies have hypothesised that landscapes could play a role either through selection by different chemical soil composition or the influence of activities which occur on different landscape types (Knapp *et al.*, 2011; Pruden *et al.*, 2012), indeed Model 2 proves this hypothesis using 23 landscape covers collected on a country-wide scale (Morton *et al.*, 2007). A positive correlation between antibiotic resistance and surrounding urban and suburban lands is likely to be due to industrial and domestic activity impacting on the rivers. The effect of neutral grassland (often comprised of dry hay meadows and pastures) had a strong association with rainfall, with an increase in rainfall leading to an increase in antibiotic-resistance genes. During periods of high rainfall, the majority of runoff from farming activities are carried in to rivers (Chee-Sanford *et al.*, 2001; Byrne-Bailey *et al.*, 2009). Landscape impacts will likely depend on the degree of runoff, the biological load in the runoff, edaphic factors and activities on the surrounding lands, which will be dictated by whether the landscapes are urban, rural or agricultural. Other landscape types may indirectly lead to an increase in antibiotic-resistance levels, for example, freshwater (classification of landscape by the LCM2007) will lead to an increase in class 1 integron prevalence by increased circulation of water bodies.

Water quality parameters that are routinely collected by several water and environmental agencies provide an attractive option for parameterising models due to the ease of widespread implementation (Bowes *et al.*, 2011). Analyses revealed that Cu, P, B, Cl, K, Na, Zn and SO_4_ levels were all correlated with each other, suggesting a mutual source. Both B and Na have previously been described as markers for WWTP effluent for approximately two decades due to their presence in effluent derived from pollutants such as detergents. Therefore, it is likely that these indicators are primarily being introduced to the site by WWTP effluent (Neal *et al.*, 2010). B and Na also correlate with class 1 integron prevalence, demonstrating clear agreement between models 2 and 3, that is, WWTP effluent is a significant driver of antibiotic resistance in the river catchment. To our knowledge, this is the first study to use water quality parameters as a predictor of antibiotic resistance load. Further work is needed to establish the precise nature of the diversity of resistance in the river sediment resistome.

In conclusion, we have generated three models, which reveal several factors influencing the environmental resistome, including previously unknown associations with surrounding land cover, temporal changes and rainfall levels. WWTPs have the ability to dramatically change antibiotic-resistance levels and ultimately determine the resistance load in rivers. Improvements in their efficacy hold the key to reducing environmental exposure, which is high in many areas, particularly during high rainfall and winter seasons. Models 2 and 3 have powerful predictive abilities and can be used as tools that predict the environmental prevalence and spread of antibiotic resistance genes (Ashbolt *et al.*, 2013).

## Figures and Tables

**Table 1:** Homogenous subsets revealed sites, which share common integron prevalence in the River Thames catchment in Oxfordshire (UK)

*Sites*	*Number of observations*	*Log integron prevalence*				
		*Subset for alpha = 0.05*				
		*1*	*2*	*3*	*4*	*5*
TC8	4	−1.7146				
TC3	3	−1.5065	−1.5065			
TC12	4	−1.3024	−1.3024	−1.3024		
TC19	2	−1.1366	−1.1366	−1.1366	−1.1366	
TC2	4	−0.9272	−0.9272	−0.9272	−0.9272	
TC18	4	−0.9115	−0.9115	−0.9115	−0.9115	
TC14	4	−0.8644	−0.8644	−0.8644	−0.8644	−0.8644
TC10	4	−0.8575	−0.8575	−0.8575	−0.8575	−0.8575
TC21	4		−0.6988	−0.6988	−0.6988	−0.6988
TC9	4			−0.5269	−0.5269	−0.5269
TC1	4			−0.3919	−0.3919	−0.3919
TC23	4				−0.3274	−0.3274
TC17	4					0.0647
Significance		0.1242	0.1804	0.0806	0.1784	0.0688

**Table 2:** Values for parameters in model 1 as determined by convergence modelling

*Treatment type*	*Value of M*
Primary (P)	Not tested
Secondary biological (SB)	0.1239
Tertiary activated sludge 2 (TA2)	0.2471
Tertiary biological 1 (TB1)	1.000
Secondary activated sludge (SA)	0.9115
Tertiary biological 2 (TB2)	0.2722
Tertiary activated sludge 1 (TA1)	0.0100
	
*Unknown variable*	*Assigned value*
**Value of S**	0.5426
**Value of X**	0.3875
**Value of C**	−1.7440

Abbreviation: WWTP, wastewater-treatment plant. **M** is the treatment type variable, **S** is rate of increase of integron prevalence with increasing WWTP impact, **X** is a parameter that defines how the impact of a WWTP decays with distance to the sampling site and **C** is a constant describing the indigenous level of antibiotic resistance in soils. A full definition of each treatment type can be found in Supplementary Information.

**Table 3:** Summary of terms and coefficients in Model 2

*Parameter*	*Coefficient*	*Standard error*	*t-Value*	*Significance level*
Constant	−0.778	0.305	−2.55	0.018
*R* (Total impact of WWTPs)	0.3207	0.0723	4.43	<0.001
Coniferous woodland	1.748	0.711	2.46	0.022
Rough grassland	−1.272	0.416	−3.05	0.006
Neutral grassland	−0.478	0.190	−2.51	0.020
Acid grassland	8.29	3.36	2.47	0.022
Heather grassland	−7.77	5.76	−1.35	0.191
Inland rock	1.476	0.461	3.21	0.004
Urban	−1.771	0.503	−3.52	0.002
Suburban	0.160	0.159	1.01	0.326
Coniferous woodland.rainfall	−1.41	1.15	−1.22	0.234
Neutral grassland.rainfall	0.994	0.386	2.58	0.017
Acid grassland.season 2	5.24	3.99	1.31	0.203
Acid grassland.season 3	7.91	4.33	1.83	0.081
Acid grassland.season 4	−8.64	4.53	−1.91	0.069
Heather grassland.season 2	−11.38	6.55	−1.74	0.097
Heather grassland.season 3	−18.70	7.60	−2.46	0.022
Heather grassland.season 4	13.37	7.84	1.71	0.102
Inland rock.season 2	−0.321	0.514	−0.62	0.539
Inland rock.season 3	1.607	0.599	2.68	0.014
Inland rock.season 4	−1.538	0.614	−2.50	0.020
Urban.season 2	1.174	0.684	1.72	0.100
Urban.season 3	3.370	0.810	4.16	<0.001
Urban.season 4	2.323	0.846	2.75	0.012
Suburban.season 2	0.046	0.178	0.26	0.798
Suburban.season 3	−0.822	0.217	−3.79	0.001
Suburban.season 4	−0.218	0.235	−0.93	0.365

Abbreviation: WWTP, wastewater-treatment plant. Summary of terms and coefficients in Model 2 alongside *t*-test values and significance values. ‘Land cover' refers to the corresponding logged percentage of land cover previously extracted (Supplementary Table 1). ‘.' indicates multiplication of variable by grouping factor or other variable. Rainfall refers to the log of the precipitation the previous day in mm. Season 1 (initial values) relates to samples from May, season 2 to samples from August, season 3 to samples from November and season 4 to samples from February.
