# Supplementary material for: Validated predictive modelling of the environmental resistome
Source: ISME J. 2015 Feb 13;9(6):1467–76. doi: 10.1038/ismej.2014.237 (PMC4438333; doi:10.1038/ismej.2014.237)
Supplement: Supplementary Information 1 [file ismej2014237x1.doc]

Supplementary guidance

Supplementary Figure 1: Extended data Figure 2: Maps from all sites (excluding TC8 which has no WWTPs in a 10 km radius) illustrating distances from all WWTPs within a 10 km buffer which drained into the sampling site, TC2 (a), TC3 (b), TC9 (c), TC10 (d), TC12 (e), TC14 (f), TC17 (g), TC18 (h), TC19 (i), TC21 (j), TC23 (k). = Sample site. = Route from river to WWTP. = Main rivers in Thames basin. = River Thames. = Tributaries in Thames River basin. = Upstream WWTP, type Secondary Biological. = Upstream WWTP, type Tertiary Biological 1. = Upstream WWTP, Tertiary Biological 2. = Upstream WWTP, Secondary Activated. = Upstream WWTP, type Tertiary Activated 1. = Upstream WWTP, type Tertiary Activated 2 (TA2). Sizes of Upstream WWTPs; 10 – 90 PE, 91 – 370 PE, 371 – 1260 PE, 1261 – 10,9000 PE, 10901 – 82300 PE.

Supplementary Figure 2: Integron prevalence from influent and effluent from three different WWTPs. Cholsey a TB2, Ascot an SA, and Benson a SB. Error bars are ± S.E.M of three biological replicates.

Supplementary Table 1: Land cover percentages extracted in ArcGIS from a 2 km radius surrounding the given sample point.

Supplementary Table 2a: Pearson coefficient values for correlations between percentage land cover and integron prevalence.

Supplementary Table 2b: Significance levels from 0 for Pearson coefficient correlations between percentage land cover and integron prevalence.

Supplementary Table 3a: Pearson coefficient values for correlations between water quality parameter measurements and integron prevalence.

Supplementary Table 3b: Significance levels from 0 for Pearson coefficient correlations between water quality parameter measurements and integron prevalence.

Supplementary Table 4: Summary of terms and coefficients in Model 3 alongside *t*-test values and significance levels.
