# Supplementary material for: Validated predictive modelling of the environmental resistome
Source: ISME J. 2015 Feb 13;9(6):1467–76. doi: 10.1038/ismej.2014.237 (PMC4438333; doi:10.1038/ismej.2014.237)
Supplement: Supplementary Information 2 [file ismej2014237x2.doc]

Definition of WWTP treatment types as taken from <http://nora.nerc.ac.uk/2810/1/SCHO0308BNVO-e-e.pdf> on 27/08/2014

P (Primary):

Treatment methods which use (screening, comminution, maceration, grit and detritus removal, pre-aeration and grease removal and storm tanks, plus primary sedimentation. Chemicals may be added such as Clariflow).

SAS (Secondary activated sludge):

Includes primary treatment as well as activated sludge treatment (including diffused air aeration, coarse bubble aeration, mechanical aeration, oxygen injection, submerged filters) and other equivalent techniques including deep shaft process, extended aeration (single, double and triple ditches) and biological aerated filters as secondary treatment.

SB (Secondary biological):

Includes primary treatment as well as treatment through rotating biological contactors and biological filtration (including conventional filtration,

high rate filtration, alternating double filtration and double filtration) and root zone treatment.

TA1 (Tertiary A1):

As SAS plus a prolonged settlement in conventional lagoons or raft lagoons, irrigation over grassland, constructed wetlands, root zone treatment (where used as a tertiary stage), drum filters, microstrainers, slow sand filters, tertiary nitrifying filters, wedge wire clarifiers or Clariflow installed in humus tanks, where used as a tertiary treatment stage.

TA2 (Tertiary A2):

AS SAS treatment but whose additional methods include rapid-gravity sand filters, moving bed filters, pressure filters, nutrient control using physico-chemical and biological methods, disinfection, hard chemical oxygen demand (COD) and colour removal, where used as a tertiary treatment stage.

TB1 (Tertiary B1):

WWTPs with a secondary stage biological process whose treatment

methods include the same additional methods as listed in TA1

treatment stage.

TB2 (Tertiary B2):

WWTPs with a secondary biological process whose additional treatment methods include the same as listed in TA2.
