# Supplementary figures and images for: Validated predictive modelling of the environmental resistome
Source: ISME J. 2015 Feb 13;9(6):1467–76. doi: 10.1038/ismej.2014.237 (PMC4438333; doi:10.1038/ismej.2014.237)

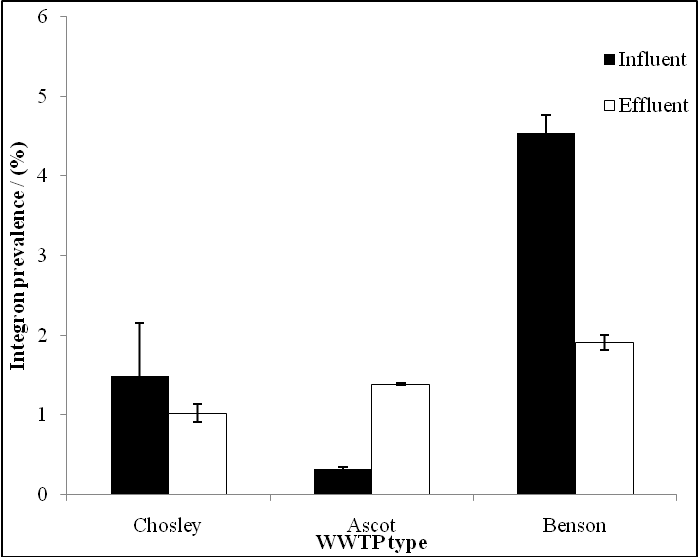

Supplement: Supplementary Figure [file ismej2014237x3.doc]

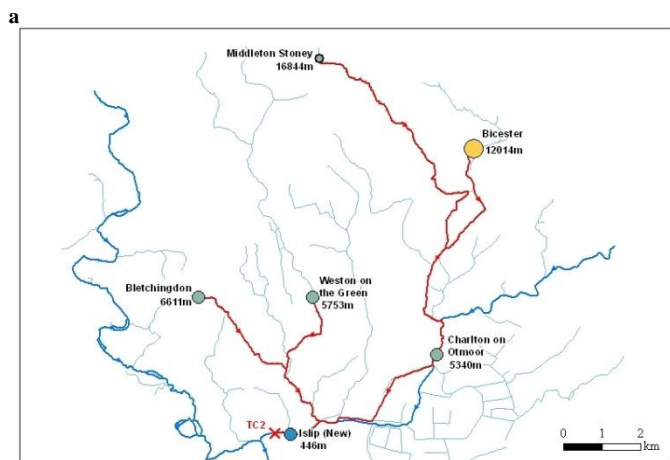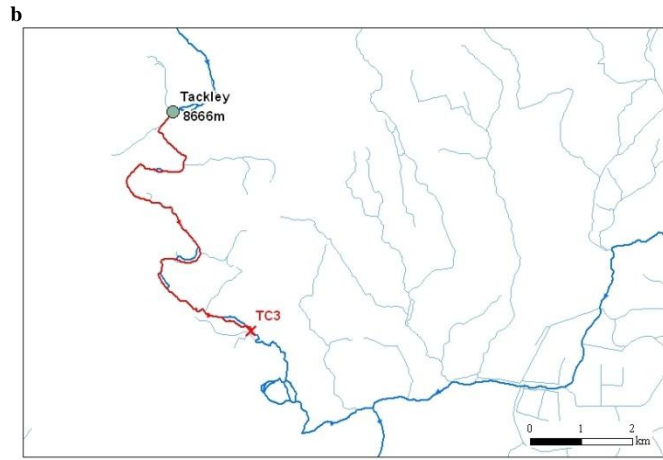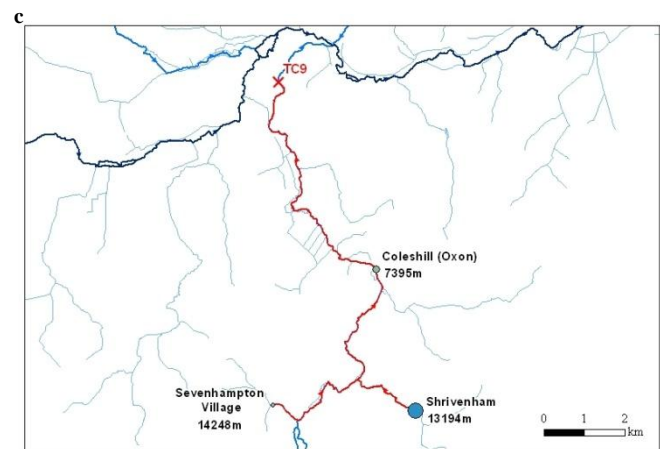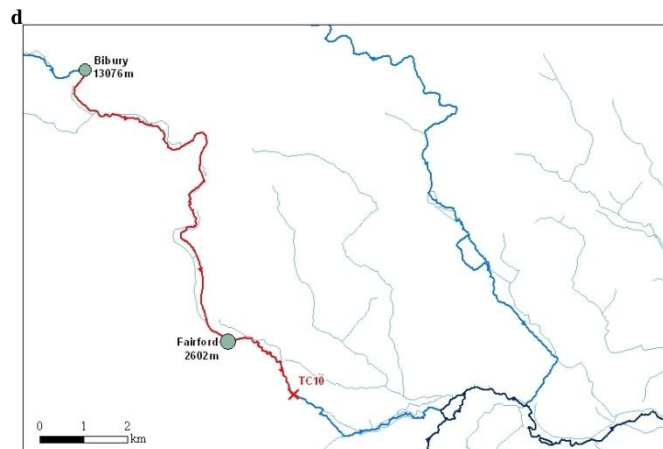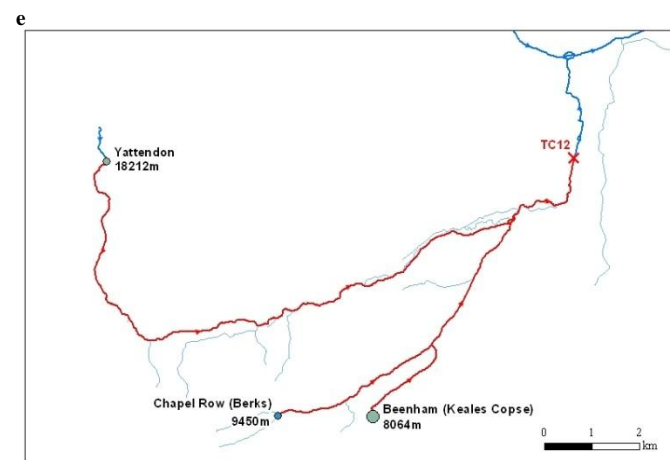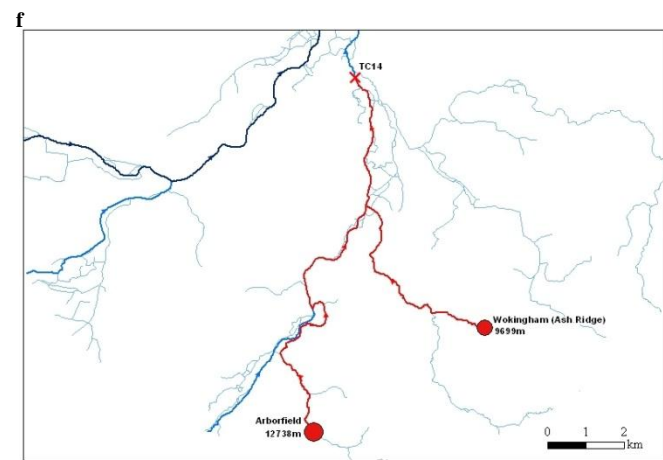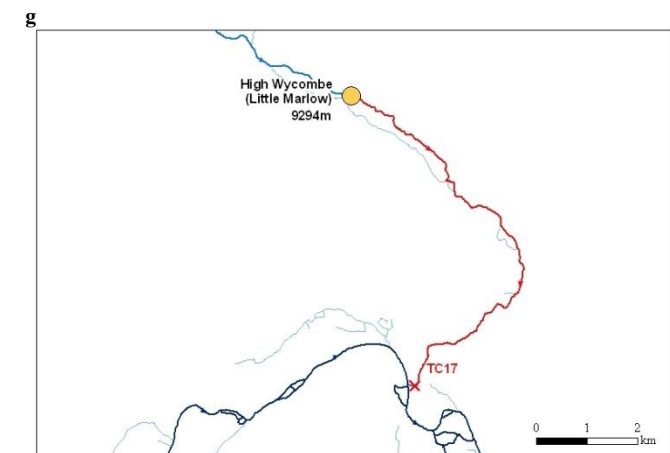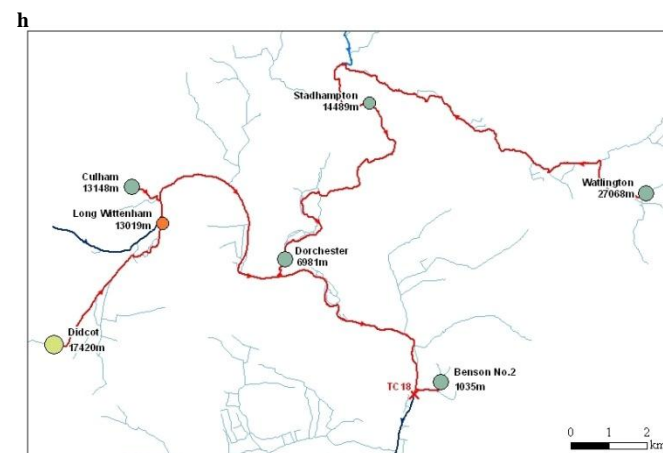

i

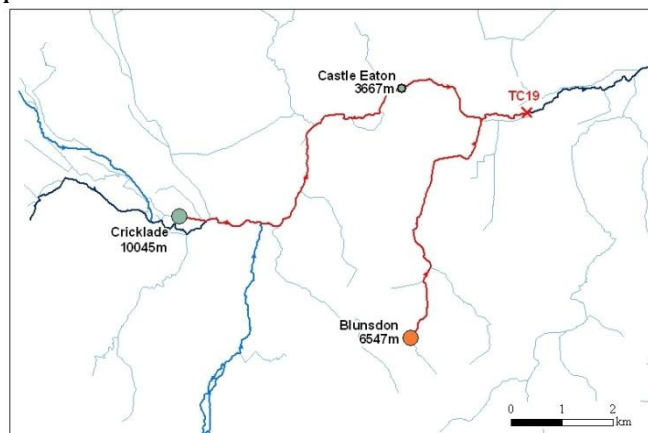

j

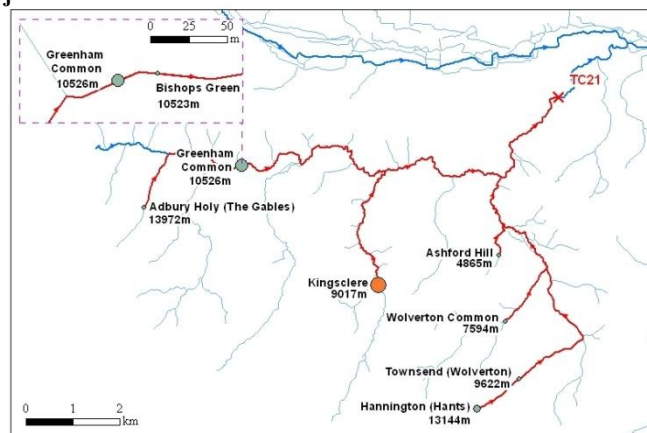

k

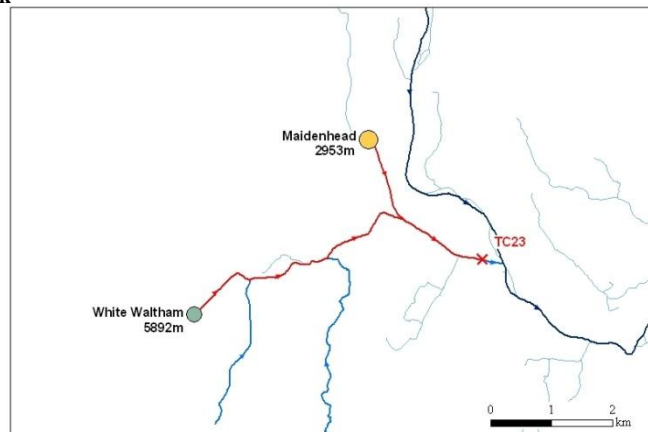

Supplement: Supplementary Figure [file ismej2014237x8.pdf]
