# Supplementary material for: Validated predictive modelling of the environmental resistome
Source: ISME J. 2015 Feb 13;9(6):1467–76. doi: 10.1038/ismej.2014.237 (PMC4438333; doi:10.1038/ismej.2014.237)
Supplement: Supplementary Table 1 [file ismej2014237x4.doc]

|  | **Land Cover percentage surrounding the sample point from a 2 km radius** | | | | | | | | | | | | |
| --- | --- | --- | --- | --- | --- | --- | --- | --- | --- | --- | --- | --- | --- |
| **Site** | **Broadleaved Woodland** | **Coniferous Woodland** | **Arable and horticulture** | **Improved Grassland** | **Rough Grassland** | **Neutral Grassland** | **Calcareous Grassland** | **Acid Grassland** | **Heather Grassland** | **Inland Rock** | **Freshwater** | **Urban** | **Suburban** |
| **TC1** | 1.55 | 0.00 | 44.74 | 36.46 | 1.73 | 7.38 | 0.40 | 0.00 | 0.00 | 1.53 | 0.00 | 0.40 | 6.21 |
| **TC2** | 0.56 | 0.00 | 60.69 | 25.96 | 3.08 | 1.93 | 0.00 | 0.00 | 0.00 | 0.00 | 0.00 | 0.00 | 7.78 |
| **TC3** | 3.62 | 0.00 | 33.96 | 46.91 | 3.50 | 3.52 | 0.00 | 0.00 | 0.00 | 0.00 | 0.95 | 0.06 | 7.48 |
| **TC8** | 2.92 | 0.00 | 52.14 | 38.91 | 4.58 | 0.00 | 0.00 | 0.00 | 0.00 | 0.18 | 0.60 | 0.18 | 0.50 |
| **TC9** | 0.64 | 0.00 | 61.73 | 32.09 | 0.84 | 3.80 | 0.00 | 0.00 | 0.00 | 0.00 | 0.90 | 0.00 | 0.02 |
| **TC10** | 2.53 | 0.00 | 46.75 | 27.67 | 4.02 | 0.00 | 0.00 | 0.00 | 0.00 | 0.90 | 8.85 | 0.40 | 8.89 |
| **TC12** | 16.49 | 1.37 | 35.73 | 34.57 | 8.67 | 0.97 | 0.00 | 0.00 | 0.00 | 0.00 | 0.00 | 0.00 | 2.19 |
| **TC14** | 4.38 | 0.00 | 35.19 | 22.66 | 1.51 | 3.86 | 0.00 | 0.00 | 0.06 | 0.52 | 3.92 | 0.74 | 27.17 |
| **TC17** | 3.05 | 0.20 | 16.79 | 41.98 | 1.83 | 3.50 | 0.06 | 0.58 | 0.26 | 0.00 | 6.39 | 2.73 | 22.64 |
| **TC18** | 2.65 | 0.00 | 42.35 | 22.20 | 2.13 | 0.00 | 0.00 | 0.00 | 0.00 | 0.00 | 2.67 | 2.69 | 25.32 |
| **TC19** | 2.97 | 0.00 | 64.47 | 13.77 | 3.07 | 12.14 | 0.00 | 0.00 | 0.00 | 2.25 | 0.00 | 1.34 | 0.00 |
| **TC21** | 9.32 | 0.24 | 62.29 | 12.74 | 5.12 | 5.68 | 0.00 | 0.00 | 0.00 | 1.02 | 2.03 | 0.00 | 1.57 |
| **TC23** | 3.04 | 1.05 | 18.02 | 32.05 | 6.23 | 0.00 | 0.00 | 0.00 | 0.20 | 7.08 | 16.15 | 1.67 | 14.50 |

**Supplementary Table 1**
