# Supplementary material for: Validated predictive modelling of the environmental resistome
Source: ISME J. 2015 Feb 13;9(6):1467–76. doi: 10.1038/ismej.2014.237 (PMC4438333; doi:10.1038/ismej.2014.237)
Supplement: Supplementary Table 2 [file ismej2014237x5.doc]

|  | **Log Integron Prevalence** | **Broadleaved Woodland** | **Coniferous Woodland** | **Arable and horticulture** | **Improved Grassland** | **Rough Grassland** | **Neutral Grassland** | **Calcareous Grassland** | **Acid Grassland** | **Heather Grassland** | **Inland Rock** | **Freshwater** | **Urban** |
| --- | --- | --- | --- | --- | --- | --- | --- | --- | --- | --- | --- | --- | --- |
| **Log integron** | - |  |  |  |  |  |  |  |  |  |  |  |  |
| **Broadleaved Woodland** | -0.2194 | - |  |  |  |  |  |  |  |  |  |  |  |
| **Coniferous Woodland** | 0.0706 | 0.6293 | - |  |  |  |  |  |  |  |  |  |  |
| **Arable and horticulture** | -0.3860 | -0.2065 | -0.4972 | - |  |  |  |  |  |  |  |  |  |
| **Improved Grassland** | -0.0108 | -0.2042 | 0.12 | -0.4848 |  |  | - |  |  |  |  |  |  |
| **Rough Grassland** | -0.3422 | 0.66 | 0.7076 | -0.1248 | -0.0572 | - |  |  |  |  |  |  |  |
| **Neutral Grassland** | 0.232 | -0.0406 | -0.2743 | 0.2206 | -0.2447 | -0.4406 | - |  |  |  |  |  |  |
| **Calcareous Grassland** | 0.3097 | -0.2214 | -0.1632 | -0.0339 | 0.2617 | -0.307 | 0.4137 | - |  |  |  |  |  |
| **Acid Grassland** | 0.4674 | -0.0068 | 0.0206 | -0.6108 | 0.3238 | -0.2414 | 0.1631 | 0.0844 | - |  |  |  |  |
| **Heather Grassland** | 0.5533 | 0.0157 | 0.3574 | -0.8912 | 0.2913 | -0.0299 | -0.0701 | -0.0199 | 0.75 | - |  |  |  |
| **Inland Rock** | 0.2792 | 0.0333 | 0.3534 | -0.2896 | -0.2127 | 0.2998 | -0.0516 | 0.1956 | -0.2213 | 0.3368 | - |  |  |
| **Freshwater** | 0.3976 | 0.0446 | 0.1795 | -0.6339 | -0.0127 | 0.0503 | -0.4209 | -0.2817 | 0.3095 | 0.6712 | 0.46 | - |  |
| **Urban** | 0.4053 | -0.0701 | 0.0373 | -0.6449 | 0.0081 | -0.2166 | -0.2021 | 0.0256 | 0.5347 | 0.6702 | 0.2415 | 0.5807 | - |
| **Suburban** | 0.3222 | -0.0302 | 0.0053 | -0.635 | 0.1199 | -0.1504 | -0.2273 | 0.0827 | 0.3382 | 0.5071 | 0.0867 | 0.5671 | 0.6371 |

**Supplementary Table 2a**

|  | **Log Integron Prevalence** | **Broadleaved Woodland** | **Coniferous Woodland** | **Arable and horticulture** | **Improved Grassland** | **Rough Grassland** | **Neutral Grassland** | **Calcareous Grassland** | **Acid Grassland** | **Heather Grassland** | **Inland Rock** | **Freshwater** | **Urban** |
| --- | --- | --- | --- | --- | --- | --- | --- | --- | --- | --- | --- | --- | --- |
| **Log integron** |  |  |  |  |  |  |  |  |  |  |  |  |  |
| **Broadleaved Woodland** | 0.1298 | - |  |  |  |  |  |  |  |  |  |  |  |
| **Coniferous Woodland** | 0.6299 | <0.001 | - |  |  |  |  |  |  |  |  |  |  |
| **Arable and horticulture** | 0.0062 | 0.1547 | <0.001 |  |  | - |  |  |  |  |  |  |  |
| **Improved Grassland** | 0.9413 | 0.1594 | 0.4115 | <0.001 | - |  |  |  |  |  |  |  |  |
| **Rough Grassland** | 0.0161 | <0.001 | <0.001 | 0.3927 | 0.6963 | - |  |  |  |  |  |  |  |
| **Neutral Grassland** | 0.1087 | 0.7817 | 0.0565 | 0.1277 | 0.0901 | 0.0015 | - |  |  |  |  |  |  |
| **Calcareous Grassland** | 0.0304 | 0.1263 | 0.2626 | 0.817 | 0.0693 | 0.0319 | 0.0031 |  |  | - |  |  |  |
| **Acid Grassland** | <0.001 | 0.9632 | 0.8883 | <0.001 | 0.0232 | 0.0947 | 0.2628 | 0.5643 | - |  |  |  |  |
| **Heather Grassland** | <0.001 | 0.9145 | 0.0117 | <0.001 | 0.0423 | 0.8386 | 0.632 | 0.8919 | <0.001 | - |  |  |  |
| **Inland Rock** | 0.0521 | 0.8203 | 0.0127 | 0.0436 | 0.1423 | 0.0363 | 0.7248 | 0.1781 | 0.1264 | 0.018 | - |  |  |
| **Freshwater** | 0.0047 | 0.7608 | 0.2172 | <0.001 | 0.9311 | 0.7316 | 0.0026 | 0.0498 | 0.0304 | <0.001 | <0.001 | - |  |
| **Urban** | 0.0039 | 0.6322 | 0.799 | <0.001 | 0.9561 | 0.1349 | 0.1637 | 0.8613 | <0.001 | <0.001 | 0.0946 | <0.001 | - |
| **Suburban** | 0.0239 | 0.8368 | 0.9709 | <0.001 | 0.4117 | 0.3023 | 0.1163 | 0.572 | 0.0175 | <0.001 | 0.5536 | <0.001 | <0.001 |

**Supplementary Table 2b**
