# Supplementary material for: Validated predictive modelling of the environmental resistome
Source: ISME J. 2015 Feb 13;9(6):1467–76. doi: 10.1038/ismej.2014.237 (PMC4438333; doi:10.1038/ismej.2014.237)
Supplement: Supplementary Table 3 [file ismej2014237x6.doc]

|  | **Integron** | **Al** | **B** | **Br** | **CHL_A** | **Ca** | **Cl** | **Cu** | **F** | **Fe** | **K** | **Mg** | **Mn** | **NH4** | **NO2** | **NO3** | **Na** | **SO4** | **SRP** | **Si** | **TDN** | **TDP** | **TP** | **Zn** |
| --- | --- | --- | --- | --- | --- | --- | --- | --- | --- | --- | --- | --- | --- | --- | --- | --- | --- | --- | --- | --- | --- | --- | --- | --- |
| **Integron** | - |  |  |  |  |  |  |  |  |  |  |  |  |  |  |  |  |  |  |  |  |  |  |  |
| **Al** | 0.19 | - |  |  |  |  |  |  |  |  |  |  |  |  |  |  |  |  |  |  |  |  |  |  |
| **B** | 0.19 | 0.43 | - |  |  |  |  |  |  |  |  |  |  |  |  |  |  |  |  |  |  |  |  |  |
| **Br** | 0.17 | 0.32 | 0.75 | - |  |  |  |  |  |  |  |  |  |  |  |  |  |  |  |  |  |  |  |  |
| **CHL_A** | 0.00 | 0.01 | 0.22 | 0.11 | - |  |  |  |  |  |  |  |  |  |  |  |  |  |  |  |  |  |  |  |
| **Ca** | 0.02 | -0.33 | 0.19 | -0.01 | 0.04 | - |  |  |  |  |  |  |  |  |  |  |  |  |  |  |  |  |  |  |
| **Cl** | 0.25 | 0.50 | 0.92 | 0.70 | 0.00 | 0.12 | - |  |  |  |  |  |  |  |  |  |  |  |  |  |  |  |  |  |
| **Cu** | 0.30 | 0.43 | 0.70 | 0.33 | 0.10 | 0.13 | 0.63 | - |  |  |  |  |  |  |  |  |  |  |  |  |  |  |  |  |
| **F** | 0.16 | 0.16 | 0.68 | 0.49 | 0.05 | 0.41 | 0.70 | 0.48 | - |  |  |  |  |  |  |  |  |  |  |  |  |  |  |  |
| **Fe** | 0.25 | 0.70 | 0.41 | 0.39 | -0.21 | -0.59 | 0.52 | 0.31 | 0.19 | - |  |  |  |  |  |  |  |  |  |  |  |  |  |  |
| **K** | 0.34 | 0.52 | 0.94 | 0.73 | 0.13 | 0.06 | 0.94 | 0.70 | 0.64 | 0.52 | - |  |  |  |  |  |  |  |  |  |  |  |  |  |
| **Mg** | -0.23 | 0.18 | 0.50 | 0.32 | -0.14 | 0.04 | 0.55 | 0.24 | 0.59 | 0.30 | 0.38 | - |  |  |  |  |  |  |  |  |  |  |  |  |
| **Mn** | 0.41 | 0.52 | 0.54 | 0.54 | 0.07 | -0.34 | 0.57 | 0.43 | 0.33 | 0.77 | 0.64 | 0.19 | - |  |  |  |  |  |  |  |  |  |  |  |
| **NH4** | 0.24 | 0.38 | 0.31 | 0.12 | -0.17 | 0.18 | 0.33 | 0.45 | 0.32 | 0.16 | 0.31 | 0.21 | 0.14 | - |  |  |  |  |  |  |  |  |  |  |
| **NO2** | 0.26 | 0.27 | 0.28 | 0.21 | 0.06 | -0.11 | 0.25 | 0.37 | 0.17 | 0.39 | 0.29 | 0.21 | 0.38 | 0.25 | - |  |  |  |  |  |  |  |  |  |
| **NO3** | 0.19 | 0.07 | 0.43 | 0.20 | -0.08 | 0.30 | 0.53 | 0.46 | 0.31 | 0.01 | 0.47 | 0.21 | 0.10 | 0.31 | 0.38 | - |  |  |  |  |  |  |  |  |
| **Na** | 0.23 | 0.48 | 0.94 | 0.73 | 0.05 | 0.12 | 0.98 | 0.63 | 0.65 | 0.47 | 0.94 | 0.52 | 0.51 | 0.34 | 0.29 | 0.56 | - |  |  |  |  |  |  |  |
| **SO4** | 0.04 | 0.23 | 0.87 | 0.69 | 0.01 | 0.32 | 0.81 | 0.60 | 0.73 | 0.26 | 0.77 | 0.65 | 0.34 | 0.30 | 0.32 | 0.43 | 0.82 | - |  |  |  |  |  |  |
| **SRP** | 0.51 | 0.49 | 0.79 | 0.60 | -0.01 | 0.15 | 0.79 | 0.65 | 0.59 | 0.48 | 0.84 | 0.28 | 0.56 | 0.47 | 0.29 | 0.32 | 0.80 | 0.65 | - |  |  |  |  |  |
| **Si** | 0.46 | 0.30 | -0.03 | -0.06 | -0.42 | -0.11 | 0.17 | 0.23 | 0.03 | 0.31 | 0.21 | -0.33 | 0.27 | 0.37 | -0.02 | 0.12 | 0.11 | -0.17 | 0.27 | - |  |  |  |  |
| **TDN** | 0.11 | 0.09 | 0.45 | 0.19 | 0.00 | 0.34 | 0.54 | 0.47 | 0.36 | -0.01 | 0.48 | 0.21 | 0.05 | 0.26 | 0.38 | 0.96 | 0.58 | 0.45 | 0.35 | 0.06 | - |  |  |  |
| **TDP** | 0.52 | 0.50 | 0.79 | 0.60 | 0.01 | 0.14 | 0.79 | 0.65 | 0.59 | 0.49 | 0.85 | 0.27 | 0.56 | 0.44 | 0.29 | 0.32 | 0.80 | 0.64 | 1.00 | 0.28 | 0.35 | - |  |  |
| **TP** | 0.52 | 0.51 | 0.83 | 0.62 | 0.16 | 0.10 | 0.81 | 0.66 | 0.60 | 0.49 | 0.88 | 0.28 | 0.58 | 0.42 | 0.29 | 0.31 | 0.82 | 0.66 | 0.97 | 0.22 | 0.34 | 0.98 | - |  |
| **Zn** | 0.33 | 0.44 | 0.80 | 0.51 | -0.02 | 0.12 | 0.80 | 0.68 | 0.44 | 0.54 | 0.80 | 0.26 | 0.51 | 0.38 | 0.36 | 0.45 | 0.78 | 0.66 | 0.76 | 0.19 | 0.47 | 0.76 | 0.75 | - |
| **pH** | -0.05 | -0.54 | -0.49 | -0.50 | 0.26 | 0.30 | -0.59 | -0.40 | -0.32 | -0.56 | -0.55 | -0.35 | -0.39 | -0.48 | -0.40 | -0.36 | -0.63 | -0.43 | -0.49 | -0.35 | -0.35 | -0.48 | -0.45 | -0.45 |

**Supplementary Table 3a**

|  | **Integron** | **Al** | **B** | **Br** | **CHL_A** | **Ca** | **Cl** | **Cu** | **F** | **Fe** | **K** | **Mg** | **Mn** | **NH4** | **NO2** | **NO3** | **Na** | **SO4** | **SRP** | **Si** | **TDN** | **TDP** | **TP** | **Zn** |
| --- | --- | --- | --- | --- | --- | --- | --- | --- | --- | --- | --- | --- | --- | --- | --- | --- | --- | --- | --- | --- | --- | --- | --- | --- |
| **Integron** | - |  |  |  |  |  |  |  |  |  |  |  |  |  |  |  |  |  |  |  |  |  |  |  |
| **Al** | 0.193 | - |  |  |  |  |  |  |  |  |  |  |  |  |  |  |  |  |  |  |  |  |  |  |
| **B** | 0.182 | 0.002 | - |  |  |  |  |  |  |  |  |  |  |  |  |  |  |  |  |  |  |  |  |  |
| **Br** | 0.247 | 0.026 | <0.001 | - |  |  |  |  |  |  |  |  |  |  |  |  |  |  |  |  |  |  |  |  |
| **CHL_A** | 0.996 | 0.952 | 0.130 | 0.438 | - |  |  |  |  |  |  |  |  |  |  |  |  |  |  |  |  |  |  |  |
| **Ca** | 0.873 | 0.020 | 0.202 | 0.938 | 0.793 | - |  |  |  |  |  |  |  |  |  |  |  |  |  |  |  |  |  |  |
| **Cl** | 0.088 | <0.001 | <0.001 | <0.001 | 0.993 | 0.396 | - |  |  |  |  |  |  |  |  |  |  |  |  |  |  |  |  |  |
| **Cu** | 0.038 | 0.002 | <0.001 | 0.020 | 0.478 | 0.374 | <0.001 | - |  |  |  |  |  |  |  |  |  |  |  |  |  |  |  |  |
| **F** | 0.261 | 0.263 | <0.001 | <0.001 | 0.708 | 0.004 | <0.001 | <0.001 | - |  |  |  |  |  |  |  |  |  |  |  |  |  |  |  |
| **Fe** | 0.079 | <0.001 | 0.004 | 0.006 | 0.153 | <0.001 | <0.001 | 0.029 | 0.203 | - |  |  |  |  |  |  |  |  |  |  |  |  |  |  |
| **K** | 0.018 | <0.001 | <0.001 | <0.001 | 0.371 | 0.698 | <0.001 | <0.001 | <0.001 | <0.001 | - |  |  |  |  |  |  |  |  |  |  |  |  |  |
| **Mg** | 0.105 | 0.226 | <0.001 | 0.025 | 0.324 | 0.762 | <0.001 | 0.104 | <0.001 | 0.038 | 0.007 | - |  |  |  |  |  |  |  |  |  |  |  |  |
| **Mn** | 0.004 | <0.001 | <0.001 | <0.001 | 0.620 | 0.019 | <0.001 | 0.002 | 0.023 | <0.001 | <0.001 | 0.183 | - |  |  |  |  |  |  |  |  |  |  |  |
| **NH4** | 0.094 | 0.008 | 0.029 | 0.406 | 0.249 | 0.215 | 0.019 | 0.001 | 0.024 | 0.259 | 0.030 | 0.153 | 0.338 | - |  |  |  |  |  |  |  |  |  |  |
| **NO2** | 0.066 | 0.062 | 0.050 | 0.148 | 0.686 | 0.471 | 0.085 | 0.009 | 0.233 | 0.006 | 0.043 | 0.151 | 0.008 | 0.082 | - |  |  |  |  |  |  |  |  |  |
| **NO3** | 0.202 | 0.637 | 0.002 | 0.171 | 0.580 | 0.035 | <0.001 | <0.001 | 0.031 | 0.940 | <0.001 | 0.152 | 0.515 | 0.032 | 0.007 | - |  |  |  |  |  |  |  |  |
| **Na** | 0.116 | <0.001 | <0.001 | <0.001 | 0.748 | 0.408 | <0.001 | <0.001 | <0.001 | <0.001 | <0.001 | <0.001 | <0.001 | 0.016 | 0.042 | <0.001 | - |  |  |  |  |  |  |  |
| **SO4** | 0.800 | 0.115 | <0.001 | <0.001 | 0.955 | 0.025 | <0.001 | <0.001 | <0.001 | 0.076 | <0.001 | <0.001 | 0.016 | 0.037 | 0.026 | 0.002 | <0.001 | - |  |  |  |  |  |  |
| **SRP** | <0.001 | <0.001 | <0.001 | <0.001 | 0.949 | 0.296 | <0.001 | <0.001 | <0.001 | <0.001 | <0.001 | 0.049 | <0.001 | <0.001 | 0.045 | 0.024 | <0.001 | <0.001 | - |  |  |  |  |  |
| **Si** | <0.001 | 0.037 | 0.846 | 0.702 | 0.003 | 0.461 | 0.246 | 0.118 | 0.840 | 0.032 | 0.141 | 0.022 | 0.058 | 0.009 | 0.867 | 0.412 | 0.435 | 0.248 | 0.061 | - |  |  |  |  |
| **TDN** | 0.455 | 0.529 | 0.001 | 0.180 | 0.988 | 0.016 | <0.001 | <0.001 | 0.011 | 0.942 | <0.001 | 0.154 | 0.713 | 0.071 | 0.008 | <0.001 | <0.001 | 0.001 | 0.015 | 0.679 | - |  |  |  |
| **TDP** | <0.001 | <0.001 | <0.001 | <0.001 | 0.920 | 0.350 | <0.001 | <0.001 | <0.001 | <0.001 | <0.001 | 0.059 | <0.001 | 0.002 | 0.046 | 0.026 | <0.001 | <0.001 | <0.001 | 0.055 | 0.014 | - |  |  |
| **TP** | <0.001 | <0.001 | <0.001 | <0.001 | 0.285 | 0.475 | <0.001 | <0.001 | <0.001 | <0.001 | <0.001 | 0.054 | <0.001 | 0.002 | 0.040 | 0.029 | <0.001 | <0.001 | <0.001 | 0.134 | 0.016 | <0.001 | - |  |
| **Zn** | 0.020 | 0.002 | <0.001 | <0.001 | 0.916 | 0.398 | <0.001 | <0.001 | 0.002 | <0.001 | <0.001 | 0.067 | <0.001 | 0.007 | 0.011 | 0.001 | <0.001 | <0.001 | <0.001 | 0.194 | <0.001 | <0.001 | <0.001 | - |
| **pH** | 0.746 | <0.001 | <0.001 | <0.001 | 0.069 | 0.039 | <0.001 | 0.004 | 0.025 | <0.001 | <0.001 | 0.015 | 0.006 | <0.001 | 0.004 | 0.011 | <0.001 | 0.002 | <0.001 | 0.013 | 0.014 | <0.001 | 0.001 | 0.001 |

**Supplementary Table 3b**
