# Supplementary material for: Validated predictive modelling of the environmental resistome
Source: ISME J. 2015 Feb 13;9(6):1467–76. doi: 10.1038/ismej.2014.237 (PMC4438333; doi:10.1038/ismej.2014.237)
Supplement: Supplementary Table 4 [file ismej2014237x7.doc]

| **Parameter** | **Coefficient** | **Standard Error** | ***t-value*** | **Significance level** |
| --- | --- | --- | --- | --- |
| **Constant** | -11.00 | 2.90 | -3.79 | <.001 |
| **Log F** | 8.56 | 2.60 | 3.29 | 0.002 |
| **Log Ca** | -3.71 | 1.12 | -3.32 | 0.002 |
| **Log K** | -2.060 | 0.629 | -3.27 | 0.002 |
| **Log Mg** | -2.101 | 0.499 | -4.21 | <.001 |
| **Log NO2** | 5.82 | 1.64 | 3.55 | 0.001 |
| **Log NO3** | 3.737 | 0.854 | 4.37 | <.001 |
| **Na** | 0.02028 | 0.00937 | 2.16 | 0.037 |
| **Log Si** | 0.615 | 0.214 | 2.87 | 0.007 |
| **Log TDN** | -3.376 | 0.843 | -4.00 | <.001 |
| **Log TP** | 1.045 | 0.220 | 4.75 | <.001 |
| **pH** | 1.636 | 0.418 | 3.91 | <.001 |

**Supplementary Table 4**
